# Luminescent Carbon Dots Synthesized by the Laser Ablation of Graphite in Polyethylenimine and Ethylenediamine

**DOI:** 10.3390/ma14040729

**Published:** 2021-02-04

**Authors:** Agata Kaczmarek, Jacek Hoffman, Jerzy Morgiel, Tomasz Mościcki, Leszek Stobiński, Zygmunt Szymański, Artur Małolepszy

**Affiliations:** 1 Institute of Fundamental Technological Research Polish Academy of Science, Pawinskiego 5B, 02-106 Warsaw, Poland; akaczmar@ippt.pan.pl (A.K.); jhoffman@ippt.pan.pl (J.H.); tmosc@ippt.pan.pl (T.M.); zszym@ippt.pan.pl (Z.S.); 2 Institute of Metallurgy and Materials Science Polish Academy of Science, Reymonta 25, 30-059 Cracow, Poland; j.morgiel@imim.pl; 3 Faculty of Chemical and Process Engineering, Warsaw University of Technology, Warynskiego 1, 00-645 Warsaw, Poland; leszek.stobinski@pw.edu.pl

**Keywords:** carbon dots, photoluminescence, laser ablation

## Abstract

Fluorescent carbon dots (CDs) synthesized by pulsed laser ablation in liquid (PLAL) are still interesting materials due to their possible applications. However, unlike CDs produced by the hydrothermal method, CDs produced the synthesis products by the PLAL method were never separated by dialysis, which differentiates the synthesis products and allows the identification of the main source of fluorescence. In this work, the synthesis of fluorescent carbon dots (CDs) was performed by nanosecond laser ablation of a graphite target immersed in polyethyleneimine (PEI) and ethylenediamine (EDA), and the synthesis products were separated by dialysis. The results of optical measurements showed that the main source of luminescence of the obtained nanostructures are fluorescent particles or quasi-molecular fluorophores created in the ablation process. In the case of ablation in PEI, most of the produced molecular fluorophores are associated with carbogenic nanostructures, while in the case of EDA, free fluorescent molecules dominate.

## 1. Introduction

Fluorescent carbon nanoparticles (FCNPs), especially with diameters below 10 nm called carbon dots (CDs), are widely used for purposes of fluorescent imaging, mainly of cells and tissues [1,2] and also as biological and chemical sensors and for catalysis [2]. Most of CDs were synthetized in a chemical way, usually by hydrothermal carbonization of citric acid (CA) functionalized with various organic, usually amine-terminated compounds like ethylenediamine (EDA) [3,4,5], branched polyethylenimine (BPEI) [6], and L- cysteine [7], because the synthesis resulted in nitrogen-doped CDs with high photoluminescence quantum yield (PLQY) [3,4,5,6,7,8,9].

There is general consensus that the CD structure consists of a carbon core, usually amorphous with crystalline domains, in different forms of hybridization, with some defects in its edge introduced by heteroatoms like oxygen or nitrogen atoms, and various functional groups or fluorescent molecules attached to the core surface, called surface and molecular states, respectively. The dispute concerned the most important source of photoluminescence. The origin of the photoluminescence spectra of carbon nanodots is a very important issue and has therefore been studied in several papers from the last decade [3,4,5,6,7,8,9,10,11,12,13,14,15]. Most of papers assigned the absorption peaks in the ultra-violet (UV) region below 280 nm to π-π* transitions of the C=C bonds within conjugated fragments in the carbon core, while the characteristic absorption peak located at 320–350 nm was assigned to n-π* transitions of the C=O bonds at the CDs edge [3,8,9]. A characteristic feature of the amino-functionalized CDs is high quantum yield photoluminescence with remarkable peaks located at 420–460 nm, induced by n-π* transitions. The high PLQY has been attributed to the doping of carbon nanoparticles with oxygen and nitrogen heteroatoms [3,4,5,6,7,8,9].

However, for several years there has been increasing evidence that the main source of light are fluorescent molecules [10,11,12,13,14,15]. Song et al. [13], analyzed CDs with very high PLQY produced from citric acid (CA) and ethylenediamine (EDA) in a typical hydrothermal process. The diagnostics applied included high resolution mass spectra (HRMS) and a nuclear magnetic resonance (1H NMR). After separation of the synthesis products through a dialysis bag of 3.5 kDa and column chromatography they appeared to consist of carbon cores, polymers and fluorescent imidazo[1,2-α]pyridine-7-carboxylic acid, 1,2,3,5-tetrahydro-5-oxo (IPCA) molecules [13,14]. Additionally, the IPCA fluorophore that passed through the dialysis bag, and not the CDs, was the main source of blue photoluminescence characteristic of the synthesis products. The optical properties of pure IPCA, such as absorbance and photoluminescence, were tested and found to correspond to those of unpurified CDs. Separation by column chromatography showed that even the nano-structures obtained at 140 °C are still mixtures of IPCA, oligomers and carbon cores. The quantum yield (QY) of specific batches changed from 0.87 to 0.09, decreasing as the IPCA content decreased.

Heating a powder mixture of citric acid and L-cysteine in a beaker for 2 h at 150 °C Shi et al. [15] synthesized two organic fluorophores, 5-oxo-3,5-dihydro-2H-thiazolo [3,2-α] pyridine-3,7-dicarboxylic acid (TPDCA) and 5-oxo-3,5-dihydro-2H-thiazolo [3,2-α] pyridine-7-carboxylic acid (TPCA). Since the same N-S-doping precursor was previously used by Dong et al. [7] for preparation of N,S-CDs in a single step hydrothermal treatment, Shi et al., also synthesized N,S-CDs according to the method reported in [7] and analyzed the synthesis products. HRMS and NMR (1H, 13C) results revealed the presence of large amounts of TPDCA and TPCA molecules in the synthesis product. Further separation of components by dialysis proved that the high QY attributed previously to N, S–doped carbon nanoparticles [7] is in fact due to the luminescence of TPDCA and TPCA, while the actual QY of the separated CDs is very low. The QY of dialysate was 0.64, that is it was identical to N,S-CDs [7], while the QY of the contents of the 1 kDa dialysis bag (retentate) was only 0.038.

In [10] Liu et al., synthesized CDs from citric acid and diethylentriamine (DETA) in the pyrolysis process at various temperatures (180, 250, and 300 °C). At lower temperatures (Dots-180, Dots-250) only clusters of molecular fluorophores were formed with PLQY = 90% and 70%, respectively. Only in the case of Dots-300, higher temperature causes the formation of carbogenic nanoparticles and a quasi-molecular fluorophore (QMF) with QY = 23%. 

Fang et al. [11] synthesized CDs using citric acid and four different reagents containing amino groups, namely L-cysteine, ethanolamine, ethylenediamine and glycine. The obtained products were separated by dialysis through a 1 kDa bag and in each case the dominant source of fluorescence were fluorophores found in dialysates. Their PLQY ranged from 50% to 70% while the PLQY of the CDs was 5 to 6 times lower.

An overview of the pathways leading to the formation of various molecular fluorophores in hydrothermal CDs synthesis is given in a review paper by Xiong et al. [12].

All these results indicate that high PLQY prescribed previously to unpurified CDs, synthesized by the bottom-up method, is mainly due to the presence of various fluorescent molecules (fluorophores) which are not actually attached to CDs. Therefore, the whole idea that fluorescent CDs can be used for purposes of fluorescent imaging, especially in biology and medicine has been called into question. Although the results [10,13,15] showed that under certain conditions the same bottom-up method produces carbon structures apparently with a much lower PLQY than that of molecular fluorophores, this fact is a little consolation. The free molecules, although much less abundant, can still be the dominant source of fluorescence. Therefore the essential issue is to produce CDs with fluorophores bound or embedded into carbogenic structure and develop methods to distinguish these structures from the aggregates of fluorescent molecules and pure carbon dots.

In contrast to CDs synthesized by the hydrothermal method, the origin of the fluorescence of CDs synthesized by laser ablation in liquid has never been analyzed in detail. Therefore, in this work, we have synthesized CDs by pulsed laser ablation of a carbon target in ethylenediamine (EDA) and polyethylenimine (PEI). The aim of the study is to answer the question whether fluorophores are formed during the synthesis of CDs by the pulsed laser ablation in liquid (PLAL) method and whether the optical properties of the synthesis products, such as absorbance and photoluminescence, can help distinguish fluorescent CDs from aggregates of free molecular fluorophores and carbon nanoparticles. In any case, the results obtained by two very different methods may allow a better understanding of the formation of luminescent CDs. The conditions during the laser ablation in liquid are fundamentally different from slowly progressing hydrothermal treatment [16,17]. Ablated particles (ions, atoms, and atom clusters) are heated by the laser beam to temperatures of up to several kilokelvins. They interact with the surrounding liquid and undergo chemical reactions inside the cavitation bubble. During the plasma cooling phase nanoparticles are formed, diffuse into the surrounding liquid and form a colloidal solution. The entire cycle takes about 1 ms.

## 2. Materials and Method

### 2.1. Synthesis of CDots

Graphite target Goodfellow (carbon, 99.997%) irradiation was performed using an Nd:YAG laser Quantel, 981E (Lumibird, Lannion, France) operating at a wavelength of 532 nm with a 10 ns pulse duration and repetition rate of 10 Hz. The conditions of synthesis were similar to those used previously in [18]. The graphite target was immersed in a liquid-branched polyethylenimine (PEI, average Mw ~800, Merck) or ethylenediamine (EDA, p.a., absolute, ≥99.5, Merck) in a quartz beaker with a diameter of 40 mm and irradiated for 15 min with a laser fluency of 3.5 J·cm^−2^. The obtained suspension was transferred to another beaker (20 mm in diameter) and a 20–40 mm high suspension column was further irradiated, at a fluence of 7 J·cm^−2^. A small portion of the suspension labeled “raw data” was then analyzed, and the greater part was dialyzed prior to further analysis. The dialysis was made against deionized water for 5 days. After dialysis the excess water was evaporated by heating to 60 °C. Both the CDs left in the dialysis bag 0.5 kDa (retentate) and the dialysate were analyzed. However, only the dialysates collected after the first and second day of dialysis were analyzed. The ratio of photoluminescence to absorbance was similar in both cases, but the signals were much weaker after the second day of dialysis.

### 2.2. Characterization

The analysis of the synthesized nanoparticles was performed using absorption and fluorescence spectroscopy, infrared spectroscopy (FTIR) and transmission electron microscopy (TEM, HRTEM). The absorbance of carbon nanoparticles was measured with a Multiscan GO spectrophotometer (Thermo Fisher Scientific, Waltham, MA, USA) and the photoluminescence spectra were taken with the use of a fluorescence spectrometer FS 5 Edinburgh Instruments (Edinburgh Instruments, Livingston, UK). FTIR spectra were taken with the use of a Thermo Scientific Nicolet iS10 spectrometer (Thermo Fisher Scientific, Waltham, MA, USA). The images of carbon particles were taken with a high-resolution Titan Themis 200 kV G2 transmission electron microscope (FEI Technologies Inc., Hillsboro, OR, USA) with a field-emission gun operating at 200 kV.

## 3. Results

### 3.1. CDs Synthesized in PEI

**Figure 1:**
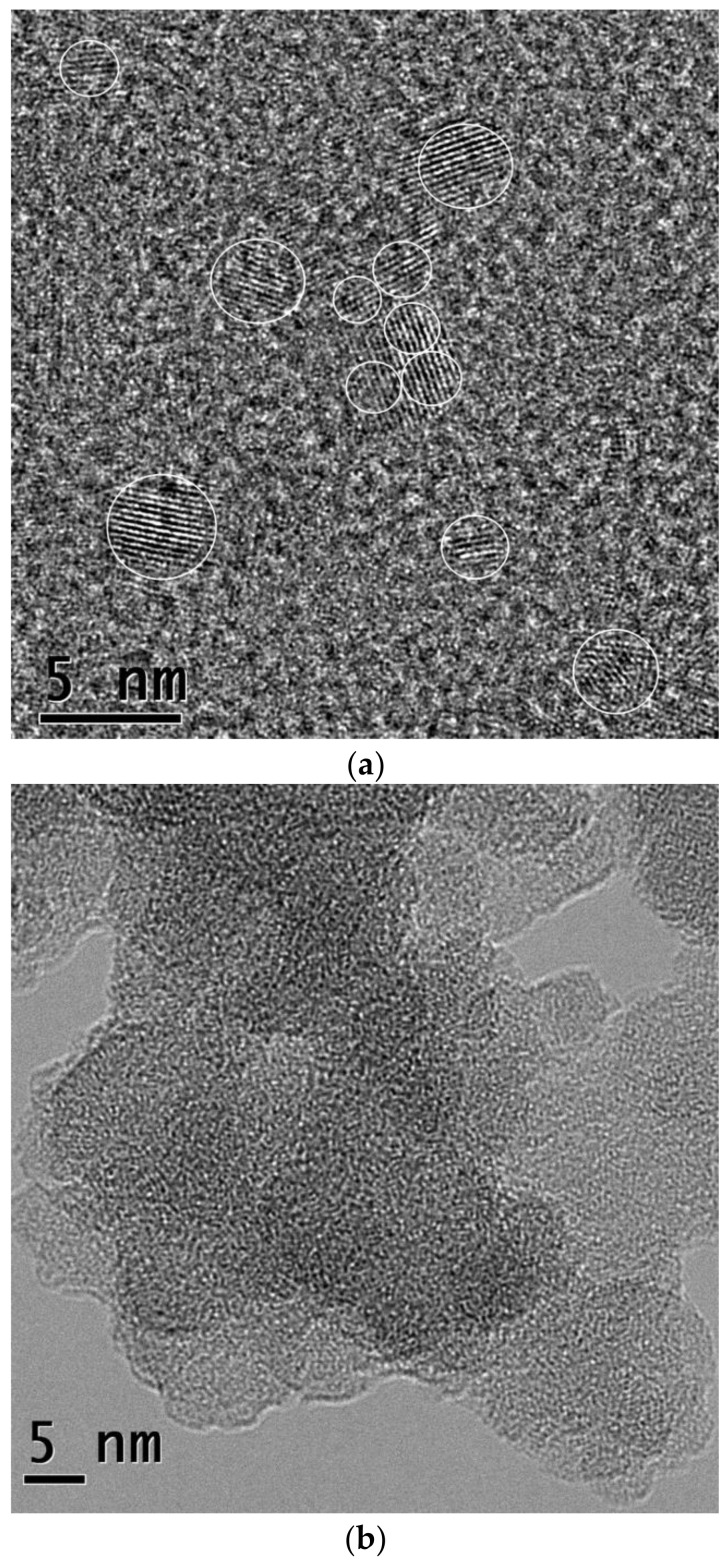
High resolution transmission electron microscopy (HRTEM) images of dialysis products synthesized in polyethyleneimine PEI: (**a**) fluorescent carbon dots (CDs) (retentate) and (**b**) dialysate.

High-resolution TEM images of the nano-structures separated by dialysis are shown in Figure 1. The CDs left in the dialysis bag have crystalline structure and a fairly spherical shape with diameters from 1 to 3 nm (Figure 1a,b). HRTEM images expose distinct crystal fringes with a lattice spacing of 0.22 nm, which corresponds to the plane (100) of the graphite lattice [19].

Unlike the contents of the dialysis bag, the dialysate contains only conglomerates of amorphous nanostructures (Figure 1b).

**Figure 2:**
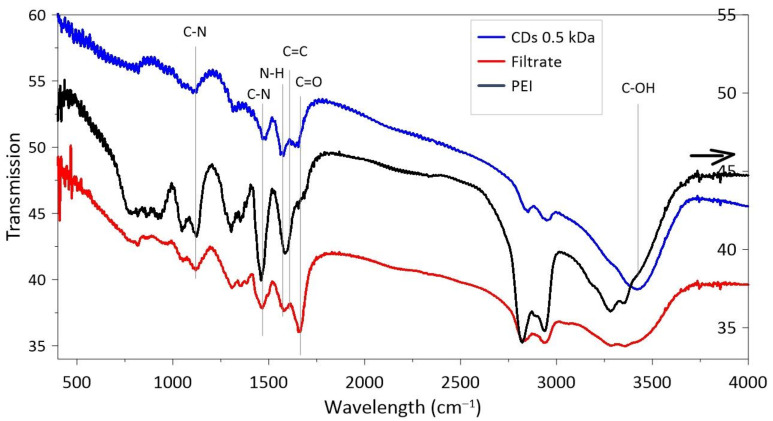
Fourier transform infrared (FTIR) spectra of nanostructures synthesized in PEI. The black arrow indicates the scale for PEI.

The FTIR spectra of CDs, dialysate and PEI are shown in Figure 2. As expected both CDs’ and dialysate’s FTIR have many characteristic absorption bands of PEI like stretching vibrations of C–N (C–NH–C) at 1126 cm^−1^, stretching vibrations of C–N bonds at 1470 cm^−1^, bending vibrations N-H at 1570 cm^−1^, and stretching vibrations of C–OH at 3423 cm^−1^.

Vibrations in the IR spectrum around 1600 cm^−1^ can be associated with C=C stretching, and around 1650 cm^−1^ with C=O of amide and carboxylic groups. The stretching vibrations of C=C and/or C=N (~1600–1610 cm^−1^) bonds confirm the presence of aromatic domains [19]. Both of these bonds are more distinct in the CDs and the dialysate than in pure PEI.

**Figure 3:**
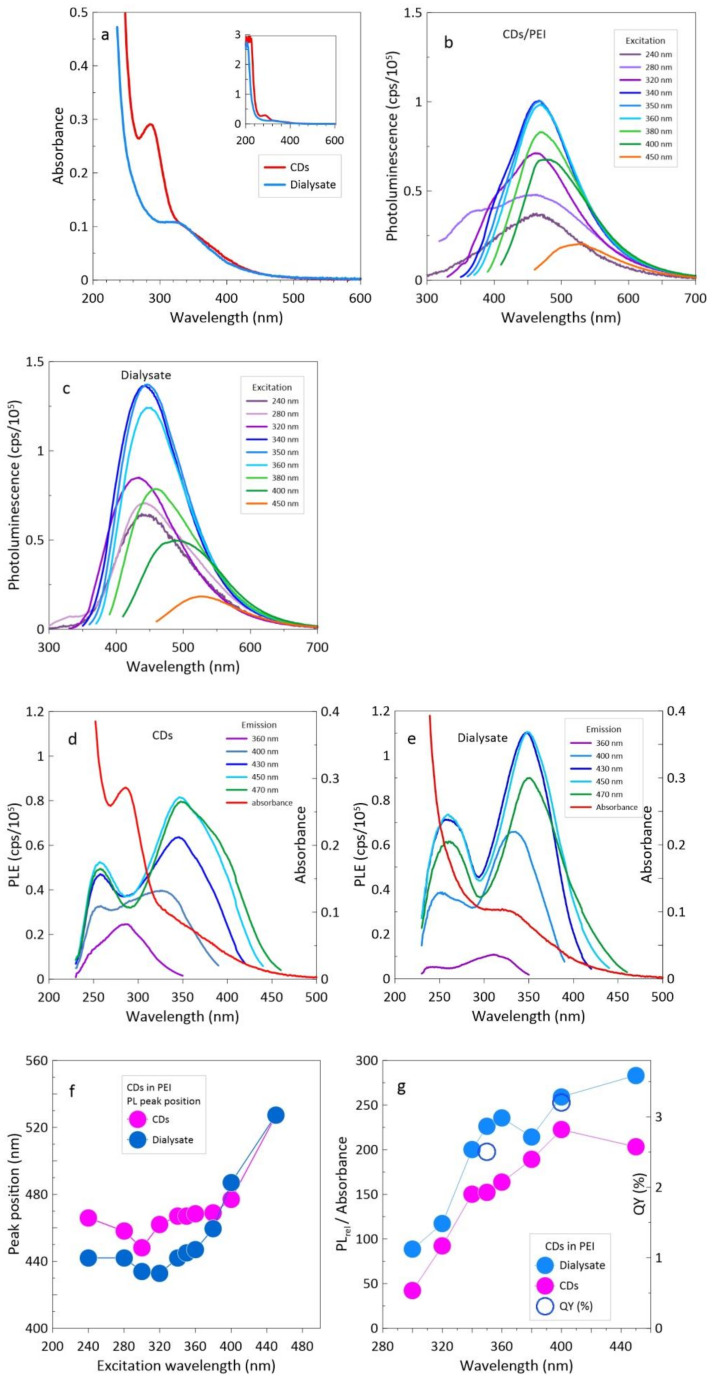
Optical properties of dialyzed CDs and dialysate: (**a**) absorbance; (**b**) PL of CDs; (**c**) PL of dialysate; (**d**) PL excitations scans of CDs; (**e**) PLE of dialysate; (**f**) PL peak position; and (**g**) ratio of integrated photoluminescence to absorbance (QYrel).

The results of optical measurements are presented in Figure 3. The absorbance of the products separated by dialysis is shown in Figure 3a. The CDs show a pronounced absorption peak at ~288 nm and a shoulder starting from ~330 nm, while the dialysate shows a broad local maximum at 330 nm. The inset shows the full spectra with additional peaks at ~215 and 205 nm for the CDs and the dialysate, respectively.

The photoluminescence (PL) of the CDs and the dialysate is shown in Figure 3b,c. Despite the significant differences in the particle structure shown by HRTEM, the differences in luminescence are rather small. The maximum of the strongest PL peaks is located at 467 nm and at 444 nm in the case of CDs and dialysate, respectively. In the case of dialysate these prominent peaks are significantly stronger than other peaks. The Stokes shift is 0.92 eV for the 467 nm fluorescence peak and 0.78 eV for the 444 nm peak (in relation to the PL excitation (PLE) peak at 347 nm), suggesting a slightly different energy level structure for CDs and the dialysate’s particles. In both cases, the CDs and the dialysate have a considerable red shift of photoluminescence peaks which is observed only at excitation with a wavelength λ ≥ 400 nm (see Figure 3f). In the case of CDs, the apparent dependence of the PL wavelength for CDs excited with λ ≤ 340 nm results from the contribution of higher energy levels corresponding to the absorbance peak at ~288 nm.

The PLE scans, which show variations of photoluminescence intensity as a function of the excitation wavelength, are presented in Figure 3d,e. In both cases, CDs and dialysate, the strongest emission in a range of 430 to 470 nm is excited with 347 nm, and somewhat weaker with 258 nm. The peaks excited by 347 nm radiation are in the case of dialysate essentially slimmer than those of the CDs. In the case of CDs, the PLE peak for the emission of 360 nm corresponds to the 288 nm absorption peak, and PLE scan for 400 nm shows the share of all three excitation peaks—258, 288 and 347 nm.

Figure 3f,g show the position of PL peaks and relative QY, defined as the ratio of integrated photoluminescence to absorbance, as a function of excitation wavelength. The QY measured for 350 and 400 nm is about 2.5% and 3.3%, respectively. The results are similar to those obtained by Bhattacharyya et al. [19] who synthesized CDs using microwave assisted pyrolysis with citric acid and branched polyethyleneimine (BPEI) as precursors.

It was widely recognized that absorption peaks between 220 and 280 nm are associated with π-π* of conjugated carbon structures in the carbonic core, while the peak at 320 nm represents the n-π* transition of the C=O bond [3,8,9,13]. Such interpretation was also consistent with calculations of Sudolská et al. [20] although the transitions their results revealed that the absorption bands above around 300 nm were dominated rather by interlayer π-π* charge transfer components rather than n-π* transitions. Similar peaks are observed in Figure 3a but they do not appear among the strongest PLE peaks (Figure 3d,e).

On the other hand PLE peaks at 258 and 347 nm correspond closely to PLE peaks obtained from organic fluorophores observed in [10,11,13,15]. The same position of the dominant PLE peaks (at 258 and 357 nm) observed in CDs and dialysate indicates that despite the different structure of their constituents, these transitions are derived from similar emitters with the same HOMO–LUMO energy levels, even if both CDs and dialysate have additional emitters with different radiative transitions. Thus, the CDs and dialysate contain similar fluorescent molecules or quasi-molecular fluorophores (QMF), and the peaks, 258 and 357 nm, should be assigned their π-π* and n-π* transitions, respectively. Since, unlike purified fluorescent molecules, whose PLE peaks closely correspond to the absorption peaks [10,13,15], both CDs and dialysate also have additional emitters, their absorbance corresponds rather to the absorbance of carbogenic nanoparticles containing some QMFs. This issue is discussed further in the discussion.

It must be emphasized that the differences between optical properties of CDs and dialysate are rather minor. These very weak changes after the 5 day dialysis process is a strong indication that the observed luminescence is due to fluorescent molecules bound to the crystalline CDs and to the amorphous nanoparticles (observed in the dialysate). Since it would be rather irresponsible to assume that all produced molecular fluorophores are bound to carbogenic structures, the dialysate may contain some free fluorescent molecules. However, the relative QYrel, defined as the ratio of integrated photoluminescence to absorbance, is not only similar for CDs and dialysate (see Figure 3g) but is also very close to the QYrel of pristine CDs before dialysis.

### 3.2. CDs in EDA

**Figure 4:**
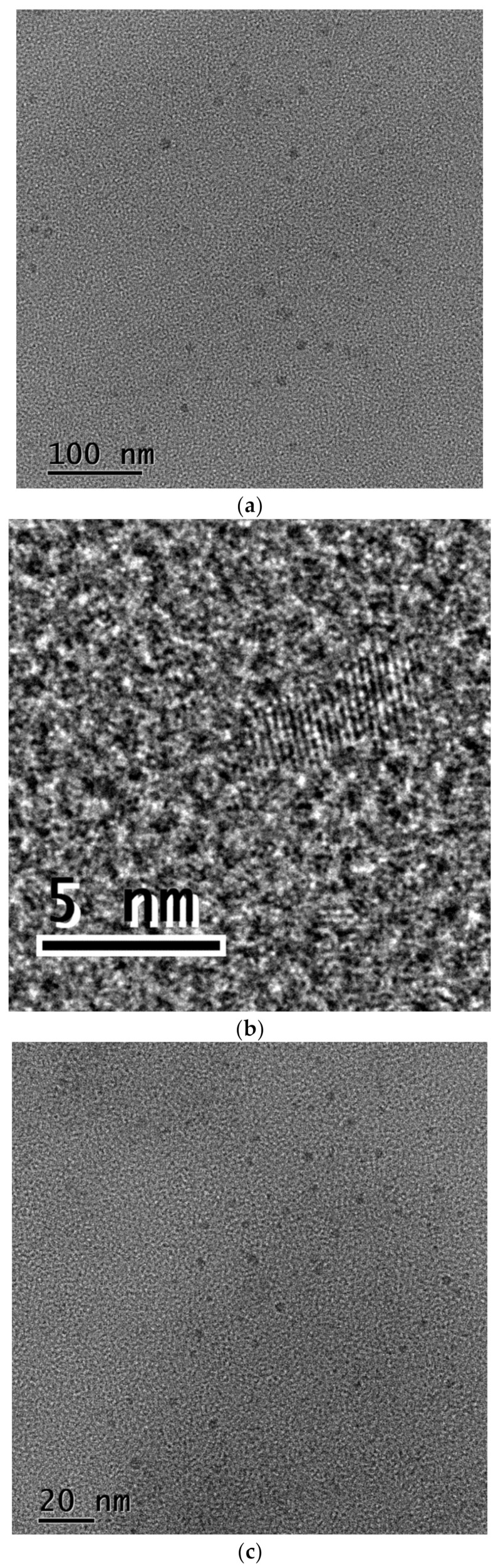

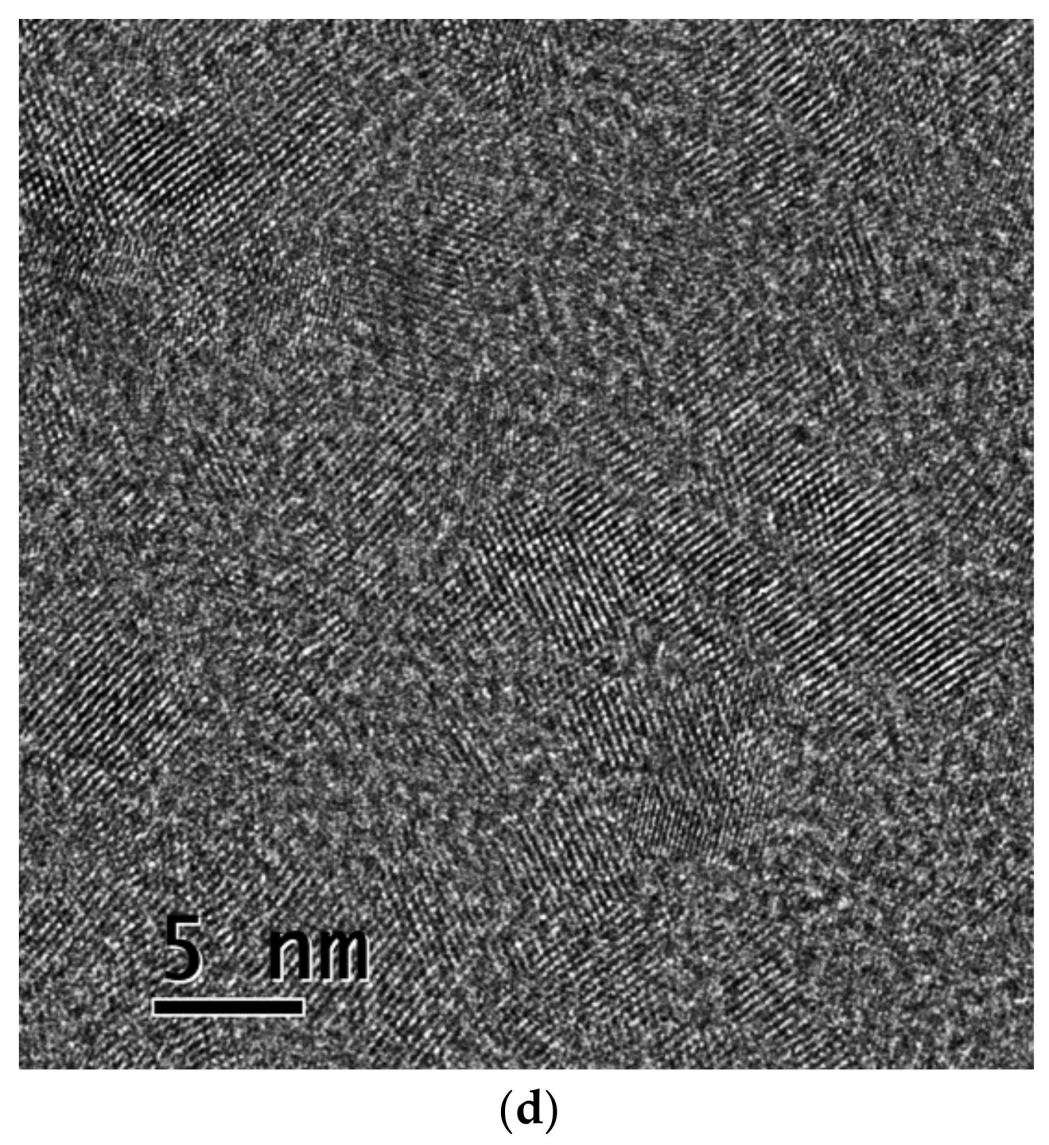
HRTEM images of dialysis products synthesized in EDA: (**a**,**b**) CDs and (**c**,**d**) dialysate.

High-resolution TEM images of the nano-structures synthesized in EDA and separated by dialysis are shown in Figure 4. Contrary to the previous results, not only the retentate but also the dialysate contains numerous small crystalline CDs with rather irregular shapes. HRTEM images expose distinct crystal fringes with a lattice spacing of 0.34 nm, which corresponds to the stable configuration of graphene layers (0.335 nm). Another observed lattice spacing of 0.24 nm corresponds to the (100) facet of graphite.

**Figure 5:**
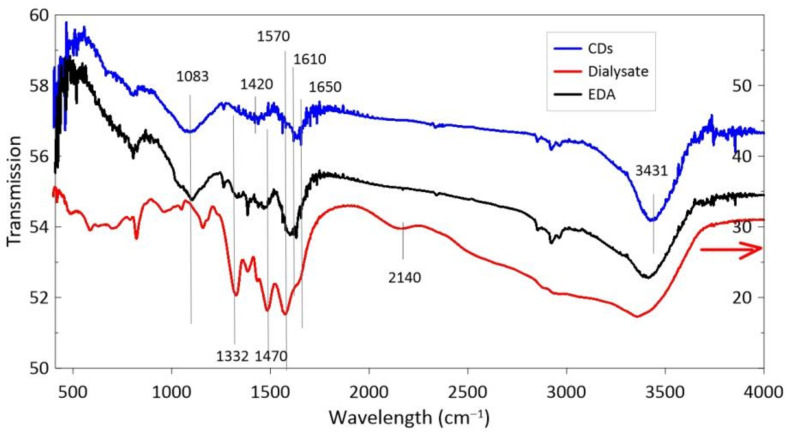
FTIR spectra of CDs synthesized in ethylenediamine (EDA). The red arrow indicates the scale for the dialysate.

The FTIR spectra CDs synthesized in EDA are shown in Figure 5. The absorption peak at 1083 cm^−1^ clearly seen in CDs is due to carboxylic groups and the next one between 1420 and 1470 cm^−1^ is due to stretching vibrations of C–N= bonds [19]. The CDs have definitely stronger vibration of the C=C band at 1600–1615 cm^−1^ than the N-H band at 1570 cm^−1^ and the C=O band at 1650 cm^−1^, in comparison to the dialysate. Both the CDs and the dialysate show a clear absorption peak related to –OH groups at about 3431 cm^−1^. EDA dialysate has clearly stronger vibration due to the N-H band at 1570 nm in relation to C=O at 1650 nm compared to the CDs as well as the PEI dialysate (the more N, the stronger the fluorescence).

**Figure 6:**
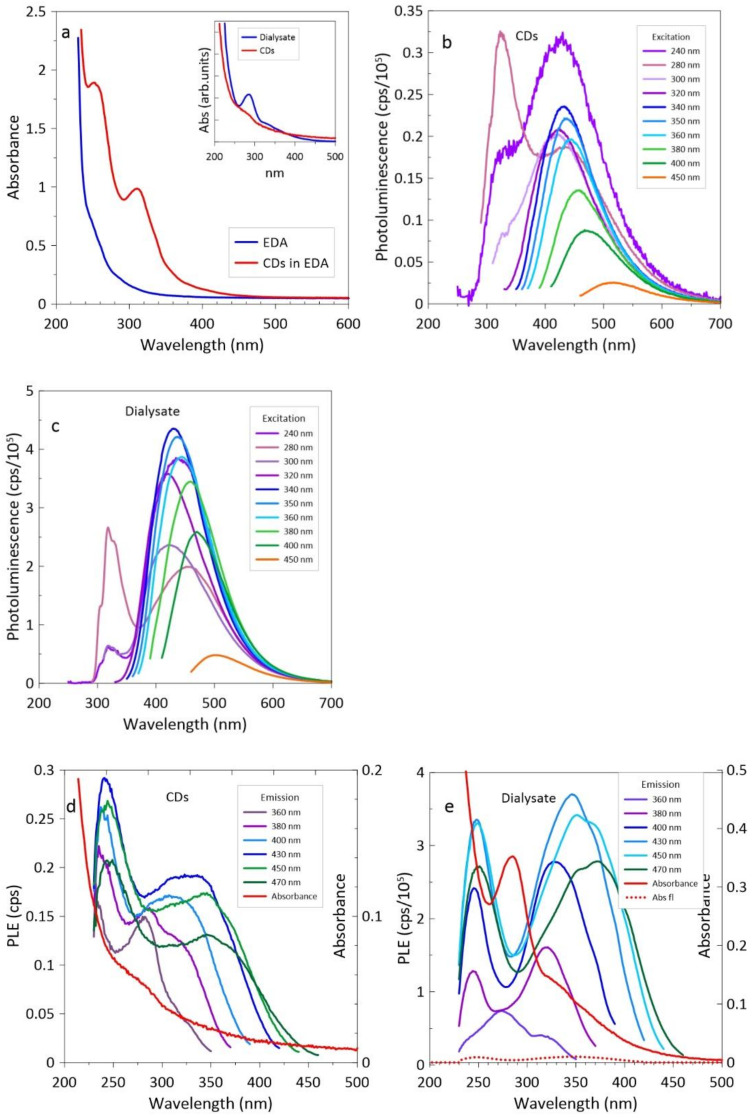

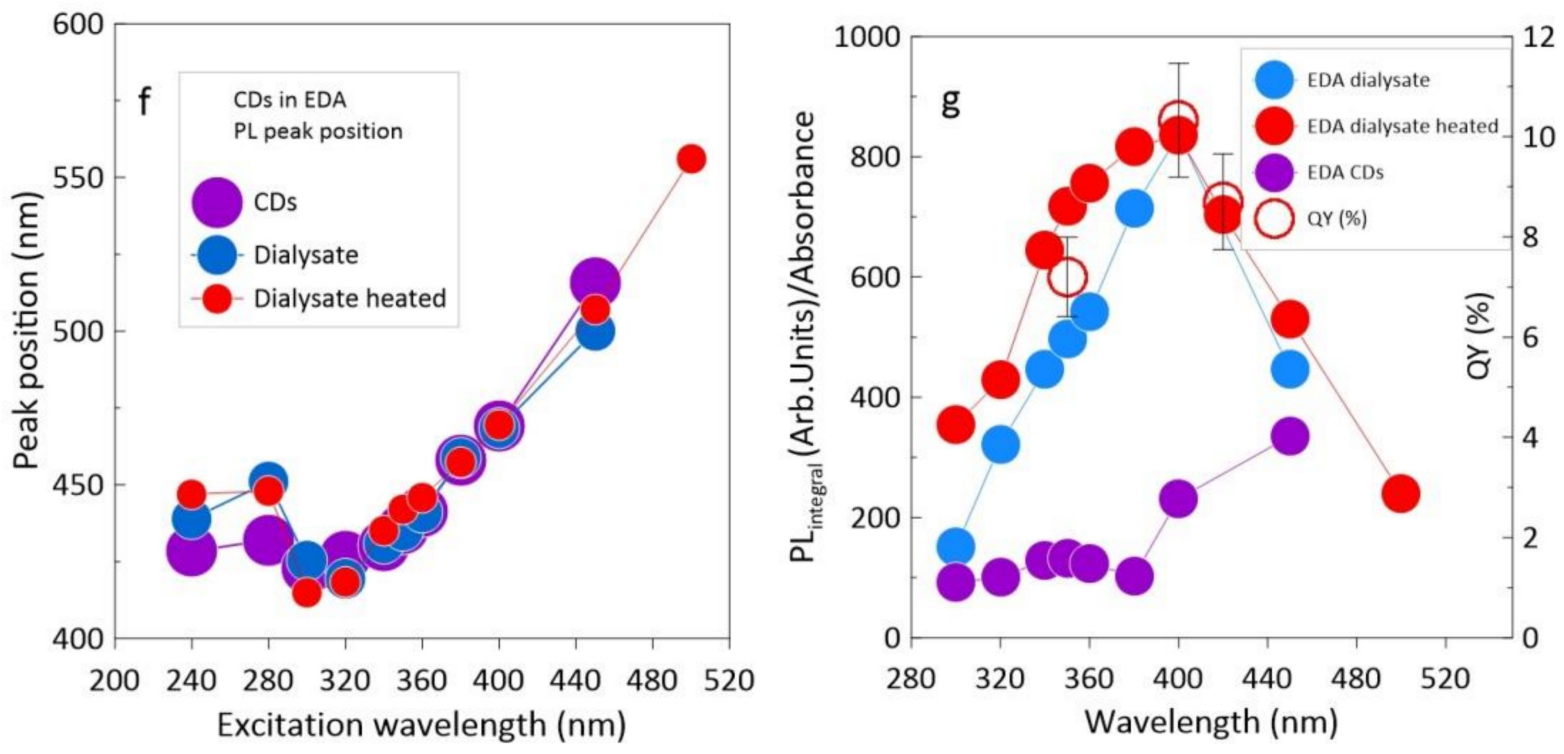
Optical properties of dialyzed CDs and dialysate: (**a**) absorbance; (**b**) PL of CDs; (**c**) PL of dialysate; (**d**) PL excitations scans of CDs; (**e**) PLE of dialysate; (**f**) PL peak position; and (**g**) ratio of integrated photoluminescence to absorbance (QYrel).

The results of optical measurements are shown in Figure 6. The difference between absorbance of the CDots in EDA after laser irradiation and the pure EDA is significant (Figure 6a). The absorbance of the products separated by dialysis is shown in the inset. Contrary to PEI the absorption peak at ~285 nm now appears in the dialysate. The results of PL and PLE measurements are shown in Figure 6b–e. In the case of CDs the first peak of PLE scans is around 243 nm, the second peak is at 283 nm (clearly seen for the emission wavelength 360 nm), and the third one is in a region between 310 and 345 nm (depending on the emission wavelength). The influence of a weakly pronounced absorption peak at ~280 nm is here clearly seen, and the prominent PLE peaks at 243 and 345 nm are poorly resolved. In the case of dialysate the most prominent PLE peaks are located at 247 and 347 nm and are well separated. The excitation from the strong absorbance peak at 280 nm is rather weak.

The position of PL peaks and the ratio of integrated photoluminescence to absorbance (QYrel) as well as measured QY are shown in Figure 6f,g, respectively. Photoluminescence quantum yields (QY) were determined from the absolute measurements using an integrating sphere as well as using quinine sulfate (H_2_O, 0.1 N H_2_SO_4_) (QY = 54.6%) and coumarin 153 in ethanol (QY = 56%) as reference standards. It is worth noting that slight overheating of the dialysate during evaporation of excess water resulted in a significant increase in QY for excitation wavelengths shorter than 400 nm. The quantum yield of CDs was below 2% at 350 nm and reached 4% at 450 nm; fluorescence at wavelengths longer than 400 nm has usually been attributed to surface states. The highest QY of dialysate amounting to 10.4% was found at λ = 400 nm, while at λ = 420 nm QY = 8.5%.

## 4. Discussion

The analysis of the optical properties of nanostructures synthesized by EDA leads to different conclusions than in the case of PEI. A significantly ~5-fold higher QY value of the dialysate than the retentate indicates clearly the presence of free fluorescent molecules. The differences in PLE scans are also significant; the prominent peaks at 245 and 345 nm are much better resolved in the dialysate and the ratio between the peaks is significantly different than that of the CDs. The excitation-dependent position of the dialysate PL peaks (see Figure 6f) is somewhat discouraging because it is not characteristic of fluorophores, but may be due to a few different fluorescent molecules. The amount of fluorescent particles is high enough to obscure the possible luminescence of the graphene-like nanoparticles present in the dialysate.

We would also like to point out why the presence of fluorescent particles clearly marked in PLE scans is not visible in the absorbance spectra. Well separated fluorophores exhibit absorbance peaks at about 245 and 345 nm, which match the excitation peaks [10,13,15]. However, in the presence of carbogenic particles, the absorbance of fluorescent molecules (free or linked to CDs) may be overshadowed by other strong absorption bands such as peaks at ~220 and 280 nm or others that do not provide significant photoluminescence. The inconsistency between the absorbance and excitation peaks, while confusing, is by no means strange. While the excitation spectrum determines the wavelengths at which light is emitted, the absorption spectrum measures the wavelengths at which a molecule absorbs light. The energy absorbed by the excited states can be dissipated without emission. In the case of organic molecules, light is usually emitted from the lowest state (Kasha’s rule).

To answer the question whether we can recognize the presence of fluorescent particles by optical measurements, we calculated the hypothetical absorbance of the fluorophore, assuming that its shape corresponds to that of PLE for 430 nm emission, and its PLQY = 60%, which is quite modest for the fluorophore. This hypothetical absorbance is shown in Figure 6e by the dotted line. It clearly shows that, in our case, when the fluorescent particles are not very numerous, their influence on the total absorption is very small.

The low quantum yield of CDs obtained in this work is quite consistent with other results obtained with the use of a laser pulse [21] or microwave heating [19,22,23]. Habiba et al. [21], who synthesized GQDs from nickel oxide suspension in benzene by means of nanosecond laser pulses, obtained CDs with QY = 5.5%. Bhattacharyya et al. [19] synthesized CDs using microwave assisted pyrolysis with citric acid and branched polyethyleneimine (BPEI) obtained CDs as precursors with 0.5 < QY < 8, depending on the BPEI concentration. The synthesis products were purified by dialysis using a 2 kDa membrane, but the dialysate content was not analyzed. Christe et al. [23] used a citric acid and urea or EDA as precursors and received CDs with QY = 2–3%. The CDs were dialysed for 48 h (EDA) or 96 h (urea) using a 1 kD membrane, but dialysates were not analyzed. In a similar experiment, Righetto et al. [22] synthesized CDs from CA and EDA [23]. The reaction products were purified by 1 day dialysis using a 1 kDa membrane. In the excitation range 320 to 375 nm, 14–15% quantum yield of the retentate was found. Dialysate was not analyzed, however, using a unique spectroscopic technique, fluorescence correlation spectroscopy (FCS), the authors were able to conclude that this emission arises from free molecules rather than from CDs. The latter result indicates the importance of a thorough separation process.

Both the PLAL and the microwave heating method are characterized by significantly shorter times and lower average temperatures compared to the hydrothermal heating used for CDs synthesis. In laser ablation, the high temperature generated by laser heating quickly relaxes within milliseconds. This suggests that the efficient production of fluorophores may proceed by processes close to the thermodynamic equilibrium, such as slow hydrothermal heating.

## 5. Conclusions

Optical measurements confirm that fluorescent molecules or quasi-molecular fluorophores are produced by laser ablation of the carbon target in PEI and EDA, albeit in relatively small amounts, with many more molecules being produced in EDA. The strongest PLE peaks, which are observed at ~250 and ~345 nm and which are well separated in dialysates, correspond closely to PLE peaks characteristic of organic fluorophores. Molecular or quasi-molecular fluorophores dominate in PLE scans but have rather little effect on the absorption spectra, at least in the case where the fluorescent molecules are not very numerous. Then their absorption spectra are obscured by strong absorption bands of carbogenic structures which do not provide significant photoluminescence. This shows that PLE spectra play an important role in the identification of optical transitions.

The results revealed significant differences between the products synthesized in PEI and EDA. Both nanostructures synthesized in PEI, the crystalline CDs and the amorphous nanoparticles observed in the dialysate, contain quasi-molecular fluorophores embedded in carbogenic structures, although the dialysate may contain free fluorescent molecules. This conclusion is based on minor differences between optical properties of CDs and dialysate especially similar quantum yield despite 5-days dialysis. In the case of CDs synthesized in EDA, the differences the between optical properties of CDs and dialysate are significant, and 5-fold higher quantum yield of the dialysate than that of the retentate clearly indicates the presence of free fluorescent molecules. It is worth mentioning that similar results were obtained at a laser wavelength of 1064 nm fluency.

## Data Availability

The data presented in this study are available on request from the corresponding author.
